# BRCA1 Versus BRCA2 and PARP Inhibitors Efficacy in Solid Tumors:A Meta-Analysis of Randomized Controlled Trials

**DOI:** 10.3389/fonc.2021.718871

**Published:** 2021-10-28

**Authors:** Shan Li, Li Tao, Haiyun Dai, Xue Gong, Yuguo Zhuo, Hui Xiang, Yueyang Zhao, Qing Gao, Liang Deng

**Affiliations:** ^1^ Department of Gastroenterology, The First Affiliated Hospital of Chongqing Medical University, Chongqing, China; ^2^ Department of Radiology, The First Affiliated Hospital of Chongqing Medical University, Chongqing, China; ^3^ Department of Respiratory and Critical Care Medicine, The First Affiliated Hospital of Chongqing Medical University, Chongqing, China; ^4^ Department of Gynecology, The First Affiliated Hospital of Chongqing Medical University, Chongqing, China; ^5^ Center for Disease Control and Prevention of Nan’an District, the Department for Infectious Disease Prevention and Control, Chongqing, China; ^6^ Neuroscience Research Center, Chongqing Medical University, Chongqing, China

**Keywords:** PARPi, BRCA1, BRCA2, meta-analysis, progression-free survival

## Abstract

**Background:**

BRCA2 mutation has a more substantial impact on the homologous recombination and superior therapeutic response to platinum-based chemotherapy than BRCA1 mutation. Whether BRCA2-mutated patients could benefit more from PARPi than BRCA1-mutated patients remains unclear. We performed a meta-analysis to assess the efficacy difference of PARPi between BRCA1 mutation carriers and BRCA2 mutation carriers.

**Methods:**

Pubmed, Embase, and Cochrane Library were comprehensively searched for randomized controlled trials (RCTs) of PARPi that had available hazard ratios (HRs) of progression-free survival (PFS) in both BRCA1-mutated population and BRCA2-mutated population. We calculated the pooled PFS HRs and 95%CI using randomized-effect models, and the difference between the two estimates was compared by interaction test.

**Results:**

A total of 11 eligible RCTs of high quality were identified through search. Overall, 1544 BRCA1 mutation carriers and 1191 BRCA2 mutation carriers were included in the final analysis. The pooled PFS HR was 0.42 (95% CI: 0.35-0.50) in BRCA1-mutated patients who were treated with PARPi compared with patients in the control group. In BRCA2-mutated patients treated with PARPi, the pooled PFS HR compared with the control groups was 0.35 (95% CI: 0.24-0.51). The difference in efficacy of PARPi was not significant between the two subgroups (*P*_heterogeneity_ = 0.40, for interaction).

**Conclusion:**

BRCA1-mutated patients and BRCA2-mutated patients could benefit from PARPi, and the efficacy is comparable. Currently, there is no evidence that BRCA2-mutated patients would benefit more from PARPi than BRCA1-mutated patients.

**Systematic Review Registration:**

https://www.crd.york.ac.uk/PROSPERO/, identifier CRD42020214582.

## Introduction

BRCA1 and BRCA2 are two critical genes involved in DNA repair *via* homologous recombination (1, 2). Cells with mutations in BRCA1 and BRCA2 are unable to activate error-free homologous recombination to repair DNA double-strand breaks, as this can result in genomic instability and even a predisposition to malignant transformation (3, 4). Many previous studies reported BRCA mutations significantly increased the susceptibility to various cancer, including breast cancer, ovarian cancer, prostate cancer, pancreatic cancer, and colon cancer (5–8).

Interestingly, BRCA-mutated cancer cells’ ability to repair DNA damage would also be impaired by the same defect, as they would become hyperdependent on the remaining repair pathway. Therefore, BRCA-mutated individuals were more sensitive to treatments relying on the induction of DNA damage, such as platinum-based chemotherapy and radiotherapy. Clinical effects of BRCA1 and BRCA2 mutations have been analyzed commonly together, but many studies have found that compared with BRCA1 mutation carriers, BRCA2 mutation carriers were associated with improved platinum-based chemotherapy response and longer progression-free duration (18.0 months for BRCA2 vs. 12.5 months for BRCA1, *P*=0.04) (9–12). Therefore, it is becoming increasingly apparent that these mutations do not have the same effects, as some studies proved that BRCA2 might have a stronger association with homologous recombination and a hypermutator phenotype (9, 11).

Targeting DNA repair in cancer, poly (ADP-ribose) polymerase (PARP) inhibitors (PARPi) were the first clinically approved target therapy, and these promising drugs revolutionarily changed the therapeutic strategies in various cancers, including breast cancer, ovarian cancer, prostate cancer, pancreatic cancer (13–24). Similarly, accumulated evidence indicated that malignancies arising in patients with BRCA mutations are sensitive to PARPi because they have DNA repair defects, as mentioned before (25). Interestingly, in a recent trial (POLO), a favorable progression-free survival (PFS) hazard ratio (HR: 0.40, 95%CI 0.20–0.85) was seen in the BRCA1 mutated patients treated with olaparib for pancreatic cancer compared with placebo, but in BRCA2 mutated patients, the benefit was not significant (HR: 0.63, 95% CI 0.39–1.02) (22). By contrast, in another trial (Profound), the efficacy of PARPi was not significant in BRCA1 mutated carriers (HR: 0.41, 95% CI 0.13–1.39), while in BRCA2 mutated patients, the benefit was significant (HR: 0.21, 95% CI 0.13–0.32) (24). Therefore, whether patients with BRCA1 mutation and patients with BRCA2 mutation can both benefit from PARPi and whether these mutations have similar prognostic effects in the use of PARPi remained unclear and controversial.

Considering the currently high costs of PARPi ($250K per PFS life-year), it is of great importance for physicians to decide which subgroup would potentially benefit more from PARPi (26). In this scenario, we performed a meta-analysis to evaluate and compare the efficacy of treatments with PARPi in patients with BRCA1 mutation and patients with BRCA2 mutation.

## Method

We followed the Preferred Reporting Items for Systematic Reviews and Meta-analyses (PRISMA) guidelines to conduct our meta-analysis (27). Our meta-analysis has been registered on PROSPERO, and the CRD code was CRD42020214582.

### Search Strategy and Selection Criteria

We comprehensively searched PubMed, Embase, and Cochrane Library for phase 2 and 3 randomized controlled trials published from the inception of each database to October 2020 with no language restrictions. The following keywords were used: niraparib, rucaparib, talazoparib, olaparib, veliparib, (poly(ADP-ribose) polymerase inhibitors, PARP Inhibitors, and randomized controlled trial (see **Supplementary Materials, Table 1**).

To be eligible, randomized controlled trials (RCT) had to fulfill all following criteria: (1) phase 2 or 3 randomized controlled trials; (2) trials evaluating the relative efficacy of a PARPi alone or with other regimens compared with regimens that did not include a PARPi in patients with cancer; (3) studies reporting HR and 95% credible interval (CI) of PFS in both BRCA1-mutated and BRCA2-mutated subgroups, regardless of mutation types (germline or somatic). We excluded trials that: (1) were single-arm or non-randomized trials; (2) were retrospective or phase 1 trials; (3) did not present HR of PFS in both subgroups of patients with BRCA1 mutation and patients with BRCA2 mutation; (4) presented HR of PFS in two subgroups, including data from patients with both BRCA1 and BRCA2 mutations in the analysis.

Moreover, in case of trials that did not include survival subgroup analysis according to BRCA mutation status in the main text, each study’s supplement would be carefully reviewed in the process of at the full-text screening stage. Additionally, if the same trial appeared in different publications, only the most complete or updated one would be included. The above screening and selection work would be done by two independent investigators. Subsequently, any discrepancies in the study selection process were discussed and resolved by a consensus formed by all investigators involved.

### Risk of Bias Assessment and Data Extraction

Two investigators would assess the quality of potentially eligible studies according to The Cochrane Risk of Bias Tool (27). Every potentially included study would be evaluated by the following criteria: (1) randomized sequence generation; (2) allocation concealment; (3) blinding of participants, personnel; (4) blinding of outcome assessment; (5) incomplete outcome data; (6) selective outcome reporting, and (7) other sources of bias. Based on this method, each risk of bias was described as low risk, high risk, or unclear risk.

From each included publications, name of study, study phase, trial design, underlying malignancy, number of patients, line of therapy, and HR for progression according to patients’ BRCA mutation types would be collated. Again, any disagreements were discussed and resolved by the consensus formed by all investigators.

### Statistical Analysis

The primary endpoint was the progression-free survival in patients with BRCA1 mutation and patients with BRCA2 mutation measured by hazard ratios. Accordingly, we derived the hazard ratios for progression and their 95% confidence intervals from each study, separately for BRCA1-mutated patients and BRCA2-mutated patients. We assessed the heterogeneity between different trials by the Cochrane’s Q statistic and *I* ^2^ statistic. Because of the clinical heterogeneity inherent in the data, we utilized random-effect models to calculate the pooled HR of PFS in all analyses. Finally, differences in pooled HRs between the two subgroups were calculated by interaction test, described by Altman and Bland (28). Accordingly, to perform the comparison of the two estimated quantities, the two estimates should be independent, not obtained from the same individuals (28). Therefore, patients with both BRCA1 and BRCA2 mutations were excluded from the analysis. Additionally, considering the relatively small sample of BRCA1 mutation carriers included in PROfound and POLO trials, within-trial interaction (deft approach) alone might not be appropriate (22, 24, 29). Therefore, we combined both across-trial and within-trial interactions to improve the power of the interaction test (29).

Subgroup analysis was also conducted to investigate the variation of the effect of BRCA mutation status on the PARPi efficacy. We only considered subgroups including more than two studies, as different cancer types, lines of therapy, and study methodology were selected. Additionally, since the number of patients in PROfound and POLO was limited, we performed another subgroup analysis, pooling the other 9 trials (22, 24). Besides, publication bias was assessed by Egger’s test and Begg’s test (30–32). Three investigators performed the statistical analyses by STATA 12.0 and R studio (metafor package), and all the data were expressed as the combination of HR and 95% CI. Moreover, a two-tailed *P* <.05 was regarded as statistically significant in the two-tailed test.

## Results

### Search Results and Patient Characteristics

**Figure 1:**
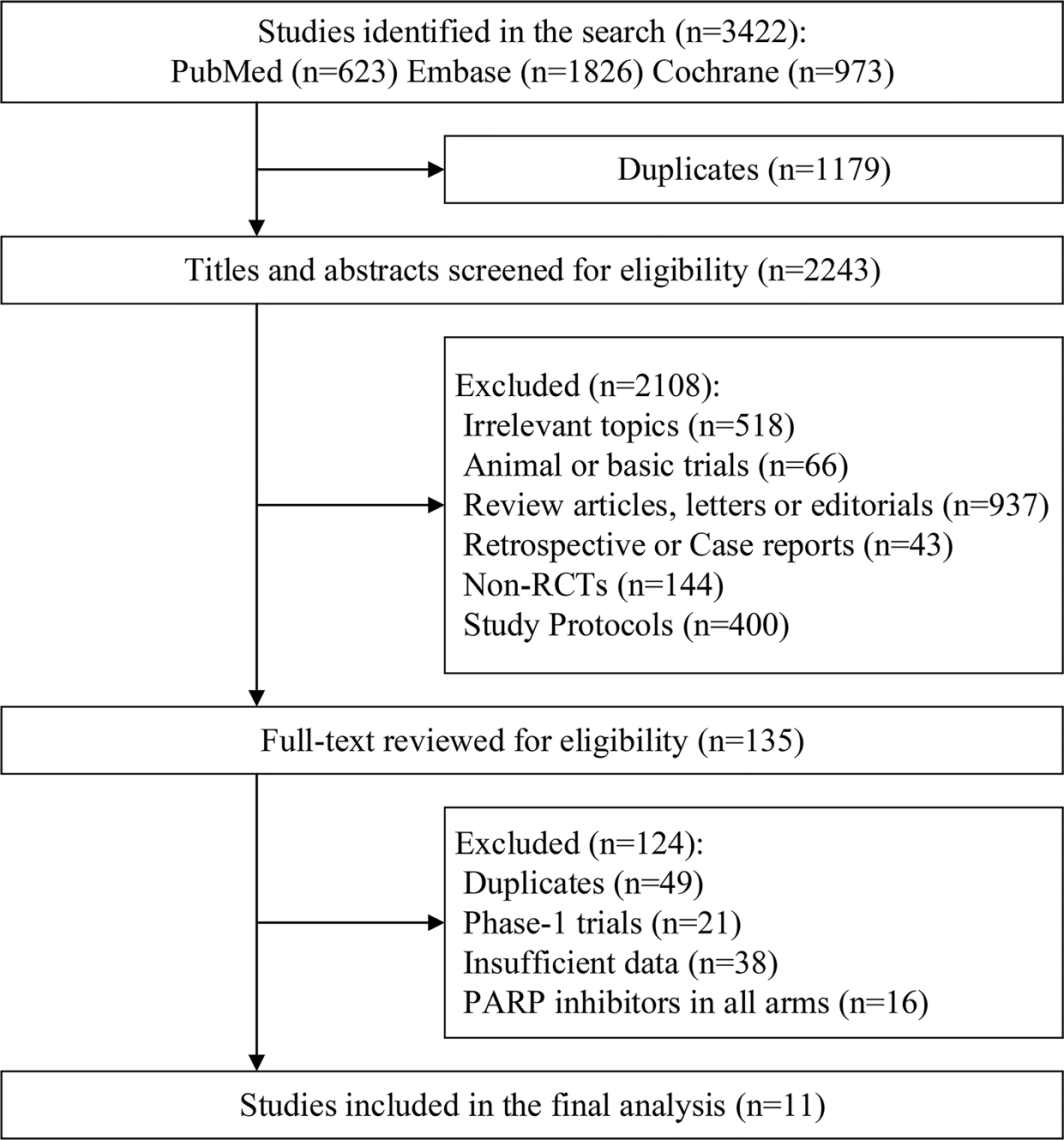
Flow diagram of study inclusion.

Initially, 3422 publications were identified through the database search. After duplication, title and abstract screen, and full-text review, a total of 11 RCT were included in the final analysis (14–24, 33) (see **Figure 1**). Additionally, we excluded Study19 to assess within-trial interaction because this trial only provided HR of PFS for patients with BRCA1 mutation while the data for BRCA2 was not available (34). At last, only one study was a phase 2 study among eligible studies, while the remaining ten trials were designed as phase 3 RCTs. All current clinical proved PARPi were included in this meta-analysis (six in olaparib, two in veliparib, and one each in talazoparib, niraparib, and rucaparib). Six trials were done in patients with ovarian cancer, three trials in patients with breast cancer, one each in pancreatic adenocarcinoma and prostate cancer.

**Table 1** listed the main characteristics of the 11 included RCTs. Overall, 2747 BRCA-deficient patients were included, of which 1544 (56.2%) were BRCA1 mutation carriers, 1191(43.4%) were BRCA2 mutation carriers, and 12 (0.4%) were patients with both mutations. The BRCA1/2 mutations were confirmed using BRCAnalysis CDx (Myriad) in ten trials, and five patients in SOLO1 with BRCA mutations which BGI confirmed, were excluded in the analysis of PFS in the SOLO1 trial (14–23). Besides, in the PROfound trial, FoundationOne CDx was utilized instead, as both BRCAnalysis CDx and FoundationOne CDx were approved by FDA for BRCA mutation testing (24, 35, 36).

**Table 1:** The basic characteristics and main outcomes of the 11 included randomized controlled trials.

Study ID	Phase	Treatment groups		Tumor types	^Ψ^Number of patients (Exp/Con)			Line of therapy	Progression-free Survival	
		Intervention	Control		BRCA1	BRCA2	Both		HR (95% CI) for BRCA1	HR (95% CI) for BRCA2
SOLO1	3	Olaparib	Placebo	ovarian cancer	191/91	66/40	3/0	Maintenance after first line	0.40 (0.29–0.56)	0.20 (0.10–0.38)
EMBRACA	3	Talazoparib	*Physician’s Choice Chemotherapy	breast cancer	133/63	154/81	0/0	≥1	0.59 (0.39–0.90)	0.47 (0.32–0.70)
VELIA/GOG-3005	3	Veliparib plus ^#^Chemotherapy followed by Veliparib	Placebo plus ^#^Chemotherapy followed by placebo	ovarian cancer	78/59	30/31	0/2	First-line + Maintenance	0.38 (0.23–0.63)	0.64 (0.27–1.56)
BROCADE	2	Veliparib plus ^#^Chemotherapy	Placebo plus ^#^Chemotherapy	breast cancer	51/53	44/46	0/0	≥1	0.745 (0.454-1.224)	0.783 (0.433-1.417)
NOVA	3	Niraparib	Placebo	ovarian cancer	84/43	50/18	1/0	Maintenance after ≥ 2 lines	0.38 (0.23-0.61)	0.12 (0.04-0.31)
SOLO2	3	Olaparib	Placebo	ovarian cancer	132/61	58/35	0/0	Maintenance after ≥ 2 lines	0.30 (0.21-0.43)	0.36 (0.22-0.62)
ARIEL3	3	Rucaparib	Placebo	ovarian cancer	80/37	50/29	0/0	Maintenance after ≥ 2 lines	0.32 (0.19–0.53)	0.12 (0.06–0.26)
OlympiAD	3	Olaparib	*Physician’s Choice Chemotherapy	breast cancer	117/51	84/46	4/0	≥1	0.54 (0.37–0.79)	0.68 (0.45–1.07)
POLO	3	Olaparib	Placebo	pancreatic cancer	29/16	62/46	1/0	Maintenance after first line	0.40 (0.20–0.85)	0.63 (0.39–1.02)
PAOLA-1	3	Olaparib plus bevacizumab	Placebo plus bevacizumab	ovarian cancer	111/49	45/31	1/0	Maintenance after first line	0.29 (0.176-0.470)	0.23 (0.090-0.541)
PROfound	3	Olaparib	Physician’s choice (enzalutamide or abiraterone)	prostate cancer	10/5	92/53	0/0	≥2	0.41 (0.13–1.39)	0.21 (0.13–0.32)

*Physician’s Choice Chemotherapy: (Capecitabine, Eribulin, Gemcitabine, or Vinorelbine) in EMBRACA, and (Capecitabine, Vinorelbine, or Eribulin) in OlympiAD; ^#^Chemotherapy: (Carboplatin and Paclitaxel) in VELIA and BROCADE; EXP, interventional arm; CON, control arm; HR, hazard ratios; CI, confidence interval; ^Ψ^Number of patients: patient with both BRCA1 and BRCA2 mutations included in “Both” group, but excluded in “BRCA1” or “BRCA2” arms and excluded in the later pooled analysis.

### Risk of Bias

The method quality of the included trials was generally moderate to good (see **Supplementary Material, Table 2**). Randomized treatment allocation sequences were generated in all trials. The main issue affecting quality was the lack of blinding because three trials (EMBRACA, OlympiAD, and PROfound) were open-labeled (15, 21, 24).

### Efficacy of PARP Inhibitors and BRCA Mutation Status

**Figure 2:**
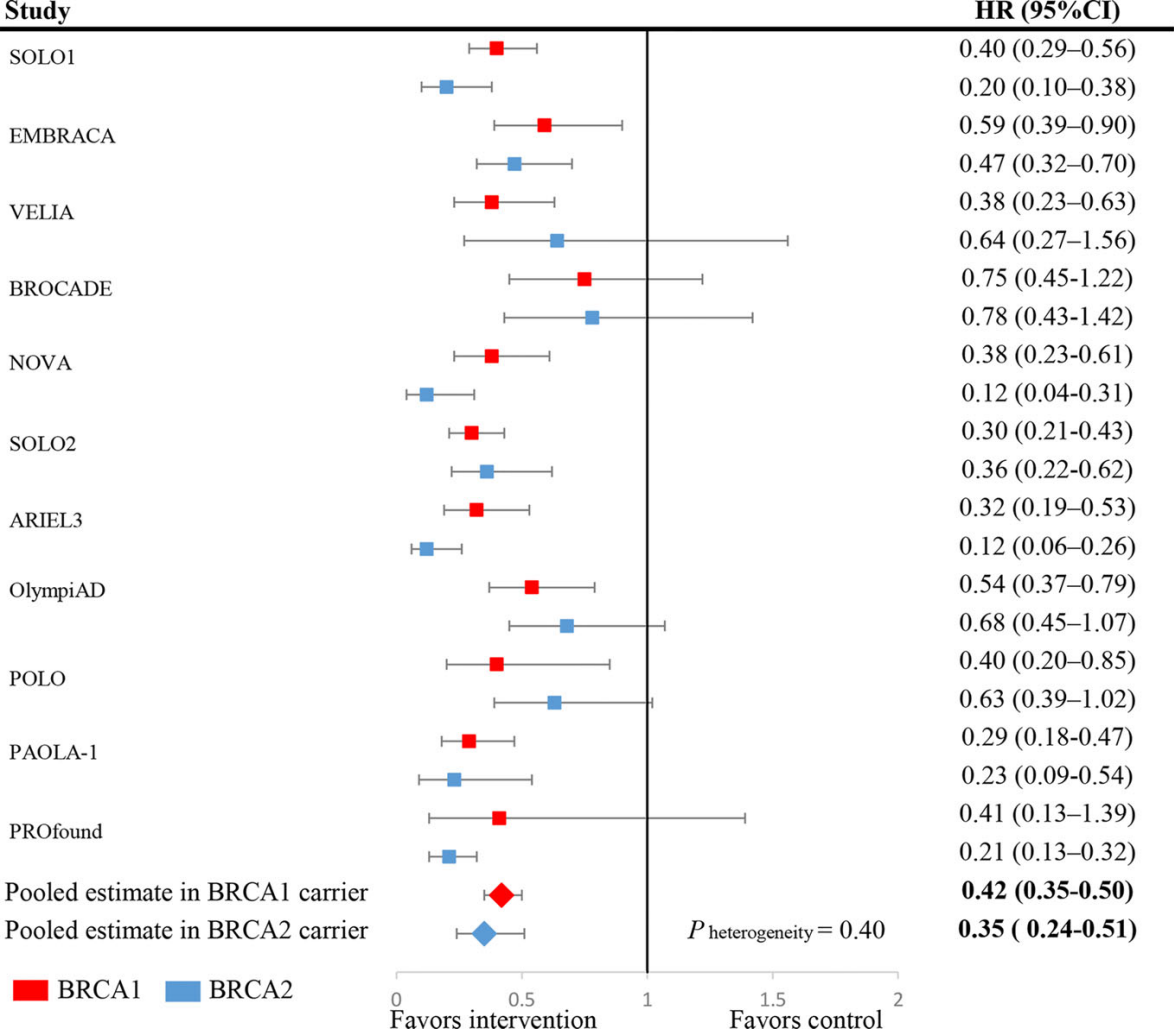
Forest plots of pooled analyses for PARP inhibitors vs. controls on progression-free survival stratified by BRCA1 mutation or BRCA2 mutation subgroups. Squares represent study-specific HRs. Horizontal lines indicate the 95% CIs. Diamonds indicate the meta-analytic pooled HRs, calculated separately in BRCA1 mutated (red) and BRCA2 mutated (blue) patients, with their corresponding 95% CIs. The p value for heterogeneity was calculated by the interaction test.

Overall, BRCA1-mutated patients who were treated with PARPi had a significantly reduced risk of progression (HR: 0.42, 95% CI: 0.35-0.50) compared with patients in the control group (see **Figure 2**). Similarly, BRCA2-mutated patients treated with PARPi were also associated with better progression-free survival than controls (HR: 0.35, 95% CI: 0.24-0.51). It should be noted that the difference in efficacy of PARPi was not significant between the two subgroups (Z=0.84, *P*_heterogeneity_ = 0.40, for interaction). Notably, patients with both BRCA1 and BRCA2 mutations were excluded in all statistical analyses to make the two estimates from BRCA1 and BRCA2 subgroups independent for the interaction test.

### Subgroup Analysis

**Figure 3:**
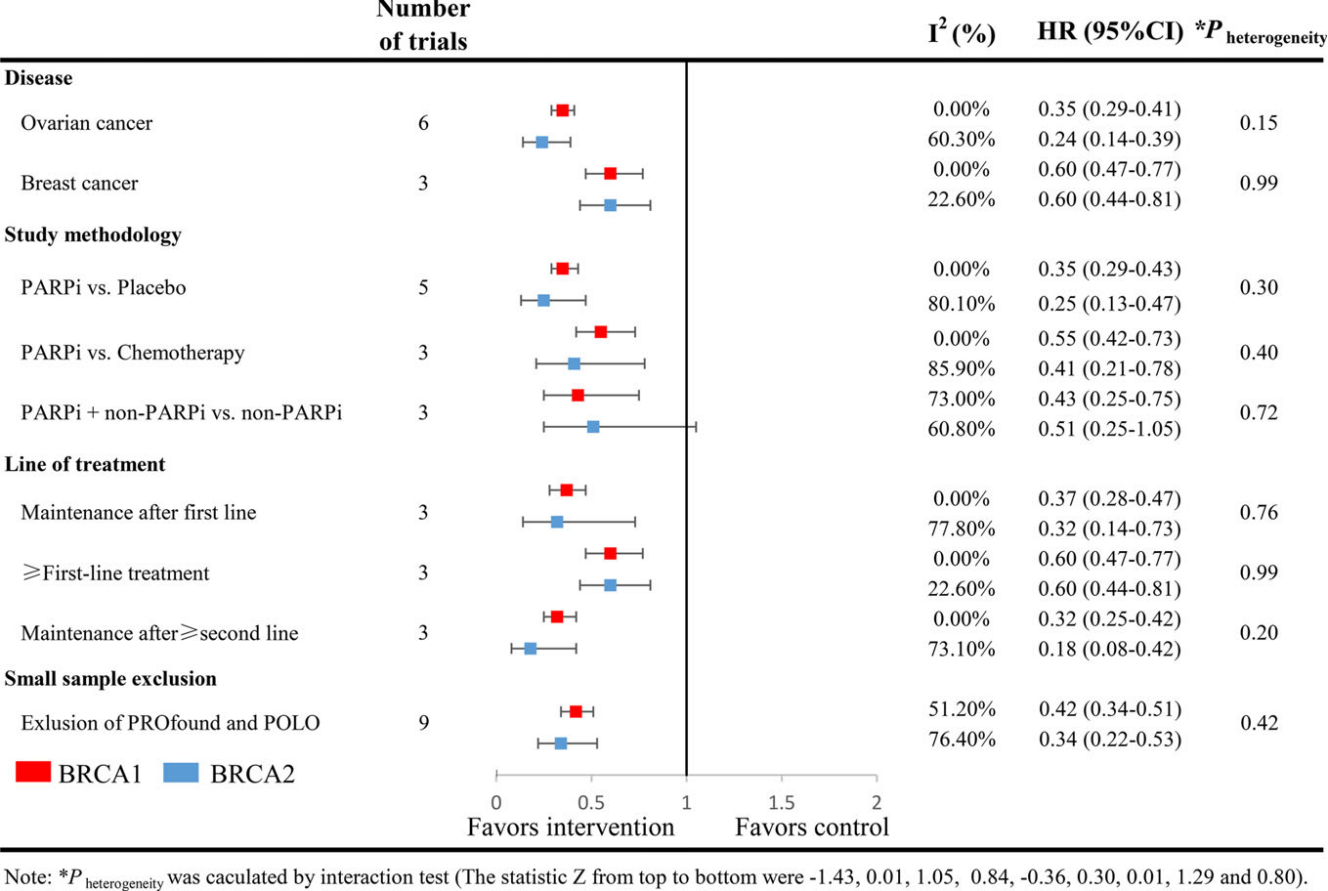
Analyses of BRCA-specific pooled PFS hazard ratios by subgroups. Squares represent subgroup-specific pooled hazard ratios (red for BRCA1 mutation carriers, blue for BRCA2 mutation carriers). Horizontal lines indicate the 95% CIs. The p value for heterogeneity is from the interaction test.

Subgroup analyses were performed according to cancer types, study methodology, and lines of the therapy. Again, no statistically significant differences in the efficacy of PARPi were found between BRCA1-mutated patients and BRCA2-mutated patients in any of these analyses (**Figure 3**). Both BRCA1 mutation carriers and BRCA2 mutation carriers could significantly benefit from PARPi regardless of cancer types and different therapeutic lines. In another subgroup analysis including solely breast cancer and ovarian cancer, excluding pancreatic and prostate cancer, a similar outcome was seen.

Interestingly, compared with BRCA2-mutated patients who were treated with chemotherapy or other standard regimens, the benefit of the addition of PARPi seemed marginal for BRCA2-mutated patients (HR: 0.51, 95%CI: 0.25-1.05). The possible explanation might be the limited number of patients involved.

### Publication Bias

The Begg’s test (*P*=0.34) and Egger’s test (*P*=0.21) showed no significant publication bias in the meta-analysis.

## DISCUSSION

To our knowledge, this study is the first meta-analysis focus on the efficacy difference of PARPi between BRCA1-mutated patients and BRCA2-mutated patients. With published data from 11 high-quality RCTs for more than 2700 patients, our pooled analysis indicated that both BRCA1 and BRCA2 mutated patients with solid tumors could benefit from PARPi, and the efficacies were comparable.

Previous studies reported that BRCA2 mutation was associated with better platinum-based chemotherapy response and a significantly longer survival time than BRCA1 mutation (10–12, 37, 38). Interestingly, these researchers believed that this divergence of therapy response might relate to the different nature of these two gene mutations. BRCA2 was directly responsible for regulating RAD51 protein, which was essential in the double-strand break repair by homologous recombination (9, 11). In contrast, besides DNA damage repair, BRCA1 was deemed as a scaffold protein and played more diverse roles, including checkpoint control and mitotic spindle assembly, as the dysfunction of BRCA1 might not necessarily affect homologous recombination (11, 39, 40). From there, the BRCA2-deficient cancer cells might be more vulnerable to DNA-damaging agents such as platinum-based chemotherapy and thereby revealing improved clinical outcomes.

Consistent with the findings from platinum-based chemotherapy, some preclinical trials studying PARP inhibition also demonstrated similar results. One preclinical study demonstrated that, although BRCA1-deficient and BRCA2-deficient cells were both sensitive to PARP inhibition, compared with wild-type cells, BRCA1-deficient cells showed a 57-fold increase in the sensitivity, while BRCA2-deficient cells indicated a 133-fold enhanced sensitivity (41).

Interestingly, our study showed a different result, in a clinical setting, that BRCA1-mutated patients and BRCA2-mutated patients could equally benefit from the treatments with PARPi. There could be several explanations. Firstly, the clinicopathological features of the two subgroups might not be the same. For instance, some studies found that among patients with ovarian cancer, compared with BRCA2 mutation carriers, BRCA1-mutated patients were diagnosed at an earlier age (11, 12). Besides, some other researches focusing on breast cancer indicated that BRCA2-associated breast cancer showed more malignant features on imaging but BRCA1-mutated counterpart showed more aggressive pathological features such as high grade (42, 43). Whether these clinical features could be the contributing factors to the efficacy of the PARPi remains unknown. Therefore, a future individual patient data meta-analysis would be highly valuable. Secondly, not all BRCA1 mutations were equal, and some of the mutation locations might result in hypomorphic BRCA1 isoforms, which could affect the efficacy of DNA-damaging agents. For example, cancer cells carrying the BRCA1(C61G) mutation or BRCA1 exon 11 respond poorly to platinum drugs and olaparib (44). On the other hand, the genetic interactions between BRCA1 and BRCA2 should also be noted (45). Some studies claimed that BRCA1 and BRCA2 might also have complementary functions, as a specific BRCA1 mutation (mutations on the C-terminal region of BRCA1) could affect the function of BRCA2 through PALB2 and result in similar clinical outcomes like BRCA2 mutation (12, 46, 47). Notably, the above evidence was all from preclinical trials. However, there could be a potential pitfall for therapeutic stratification of PARPi while all BRCA1-mutated patients continue to be considered a single clinical entity. Whether patients with such BRCA1 mutation locations could also benefit from PARPi in a clinical setting was still unclear. Hence, future studies with more detailed stratifications within the BRCA1-mutated population would be urgently needed.

Another explanation might be related to other therapeutic actions of PARPi and other functions of BRCA1 beyond DNA damage. In one recent meta-analysis studying the efficacy of PARPi in newly diagnosed ovarian cancer, a significant clinical benefit of PARPi has been obtained both in Non-BRCA mutated patients and in patients even without homologous recombination deficiency (48). In fact, besides DNA damage-induced cell death, PARP inhibitors, by inhibiting the PARP-1, can alter cellular energy metabolism and redox balance, leading to cancer cell apoptosis (49). Unlike BRCA2, which directly guided RAD51 to damage sites in the process of DNA repair, BRCA1 plays more diverse roles. Besides DNA repair, BRCA1 also played critical roles in checkpoint control, mitotic spindle assembly, sister chromatid decatenation, and centrosome duplication (9, 39). From there, the interaction between PARPi and the above functions of BRCA1 beyond DNA damage is less known and worth vast further investigation.

The major limitation of this meta-analysis was the considerable heterogeneity observed in the pooled results. It is highly likely that such heterogeneity was related to the design of the study itself, including different lines, conduction on different solid tumors, and different study methodology. Therefore, a randomized effect model was applied to take into account such heterogeneity. More importantly, numerous subgroup analyses based on the above factors were performed, and the results were consistent. No statistically significant differences in the efficacy of PARPi were found between BRCA1 mutation carriers and BRCA2 mutation carriers in any of these analyses. Secondly, an optimal treatment strategy is needed to maximize the benefit as well as minimize the risk of toxicities. However, in our study, the information regarding adverse events from the two subgroups separately was not available. The safety difference between BRCA1-mutated patients and BRCA2-mutated patients was unclear. Thirdly, since our study only extracted data at a trial level rather than an individual level, other variables, including BRCA1 mutation location, age, and different races, could possibly affect the response to the PARPi. Due to the lack of the necessary resources, we were only able to utilize the published data. Therefore, a future individual patient data meta-analysis would be warranted. In addition, the limited number of pancreatic cancer and prostate cancer might not increase the significance of this study. Although, we performed a subgroup analysis to exclude the two small sample studies and obtained still outcome. For this reason, future studies were urgently needed to verify the similar efficacy between the two groups among other malignancies. Fourthly, we could not determine overall survival (OS) because of the lack of OS data in the RCTs, which may have provided a more convincing result. From there, an updated meta-analysis would be needed in the future.

## Conclusion

Currently, there is no evidence that BRCA2-mutated patients would benefit more from PARPi than BRCA1-mutated patients. BRCA1-mutated patients and BRCA2-mutated patients could benefit from PARPi in clinical practice, and the efficacy is comparable. More clinical trials are currently warranted to verify this trend in various cancer such as pancreatic adenocarcinoma and prostate cancer.

## Data Availability Statement

The original contributions presented in the study are included in the article/**Supplementary Material**. Further inquiries can be directed to the corresponding authors.

## Author Contributions

LD and QG were in charge of funding acquisition, validation, and supervision. SL, LT, and HD performed data curation, conceptualization and methodology of the study. SL LT, HD, and XG performed data validation and formal analysis. SL, YGZ, HX, and YYZ completed the project administration, visualization and writing - original draft. SL, LT, and LD finished the manuscript writing - review & editing. All authors contributed to the article and approved the submitted version.

## Funding

This work was supported by the Joint Project Foundation of Chongqing Health Commission and Chongqing Scientific and Technological Commission (grant number 2021MSXM103).

## Acknowledgment

We want to show our gratitude and respect to Taylor Swift, Angelina Jolie, Hugh Jackman, Wang Nan, and other celebrities worldwide who share their own stories about the fight against cancer with positive energy fearlessly and who gives generous donations to cancer research. What they do could have a positive impact on the advocation of cancer prevention and emphasizes that conquering cancer is never a lonely fight, but is a war for every human being.

## Conflict of Interest

The authors declare that the research was conducted in the absence of any commercial or financial relationships that could be construed as a potential conflict of interest.

## Publisher’s Note

All claims expressed in this article are solely those of the authors and do not necessarily represent those of their affiliated organizations, or those of the publisher, the editors and the reviewers. Any product that may be evaluated in this article, or claim that may be made by its manufacturer, is not guaranteed or endorsed by the publisher.
