# Supplementary material for: BRCA1 Versus BRCA2 and PARP Inhibitors Efficacy in Solid Tumors:A Meta-Analysis of Randomized Controlled Trials
Source: Front Oncol. 2021 Oct 28;11:718871. doi: 10.3389/fonc.2021.718871 (PMC8580941; doi:10.3389/fonc.2021.718871)
Supplement: Supplementary file 1 [file Table_1.docx]

Supplementary Table 1: Search strategies in three databases

Supplementary Table 2: Risk of bias assessment

| Supplementary Table 1: Search strategies in three databases | |
| --- | --- |
| Database | Search strategies |
| Pubmed | ((niraparib OR (niraparib hydrochloride) OR Zejula OR (MK 4827) OR MK4827 OR MK-4827) OR (Rucaparib OR PF-01367338 OR Rubraca OR (AG 014699) OR AG014699 OR AG-014699) OR (talazoparib OR Talzenna OR (BMN 673) OR BMN-673 OR BMN673) OR (olaparib OR (AZD 2281) OR AZD2281 OR AZD-2281 OR AZD221 OR Lynparza) OR (veliparib OR (ABT 888) OR ABT888 OR ABT-888) OR ((Poly(ADP-ribose) Polymerase Inhibitors) OR (PARP Inhibitors) OR (Inhibitors of Poly(ADP-ribose) Polymerases) OR (Poly(ADP-ribosylation) Inhibitors) OR (Inhibitors, PARP))) AND ((randomized controlled trial [pt] OR controlled clinical trial [pt] OR randomized [tiab] OR placebo [tiab] OR clinical trials as topic [mesh: noexp] OR randomly [tiab] OR trial [ti]) NOT (animals [mh] NOT humans [mh])) AND (neoplasm OR Neoplasia OR Neoplasias OR Tumors OR Tumor OR Cancer OR Cancers OR Malignancy OR Malignancies OR Malignant Neoplasms OR Malignant Neoplasm OR (Neoplasm, Malignant) OR (Neoplasms, Malignant) OR carcinoma) |
|  |  |
| Embase | No1: ‘niraparib’ OR ‘niraparib hydrochloride’ OR ‘Zejula’ OR ‘MK 4827’ OR ‘MK4827’ OR ‘MK-4827’  No2: ‘Rucaparib’ OR ‘PF-01367338’ OR ‘Rubraca’ OR ‘AG 014699’ OR ‘AG014699’ OR ‘AG-014699’  No3: ‘talazoparib’ OR ‘Talzenna’ OR ‘BMN 673’ OR ‘BMN-673’ OR ‘BMN673’  No4: ‘olaparib’ OR ‘AZD 2281’ OR ‘AZD2281’ OR ‘AZD-2281’ OR ‘AZD221’ OR ‘Lynparza’  No5: ‘veliparib’ OR ‘ABT 888’ OR ‘ABT888’ OR ‘ABT-888’  No6: ‘Poly(ADP-ribose) Polymerase Inhibitors’ OR ‘PARP Inhibitors’ OR ‘Inhibitors of Poly(ADP-ribose) Polymerases’ OR ‘Poly(ADP-ribosylation) Inhibitors’ OR ‘Inhibitors, PARP’  No7: ‘cancer’ OR ‘carcinoma’ OR ‘malignant neoplasm’ OR ‘tumor’  No8: ‘human’  No9: 'crossover procedure':de OR 'double-blind procedure':de OR 'randomized controlled trial':de OR 'single-blind procedure':de OR (random* OR factORial* OR crossover* OR cross NEXT/1 over* OR placebo* OR doubl* NEAR/1 blind* OR singl* NEAR/1 blind* OR assign* OR allocat* OR volunteer*):de,ab,ti |
|  |  |
| Cochrane | (niraparib OR (niraparib hydrochloride) OR Zejula OR (MK 4827) OR MK4827 OR MK-4827) OR (Rucaparib OR PF-01367338 OR Rubraca OR (AG 014699) OR AG014699 OR AG-014699) OR (talazoparib OR Talzenna OR (BMN 673) OR BMN-673 OR BMN673) OR (olaparib OR (AZD 2281) OR AZD2281 OR AZD-2281 OR AZD221 OR Lynparza) OR (veliparib OR (ABT 888) OR ABT888 OR ABT-888) OR ((Poly(ADP-ribose) Polymerase Inhibitors) OR (PARP Inhibitors) OR (Inhibitors of Poly(ADP-ribose) Polymerases) OR (Poly(ADP-ribosylation) Inhibitors) OR (Inhibitors, PARP)) AND (neoplasm OR Neoplasia OR Neoplasias OR Tumors OR Tumor OR Cancer OR Cancers OR Malignancy OR Malignancies OR Malignant Neoplasms OR Malignant Neoplasm OR (Neoplasm, Malignant) OR (Neoplasms, Malignant) OR carcinoma) |

| Supplementary Table 2: Risk of bias assessment | | | | | | | |
| --- | --- | --- | --- | --- | --- | --- | --- |
| Study | Randomization | Allocation  concealment | Blinding of  participants and staff | Blinding of  outcome assessors | Incomplete outcome data | Selective reporting | Other bias |
| SOLO1 | low | low | low | low | low | low | low |
| EMBRACA | low | low | high | low | high | low | low |
| VELIA | low | low | low | low | low | low | low |
| BROCADE | low | unclear | low | unclear | low | low | low |
| NOVA | low | low | low | low | high | low | low |
| SOLO2 | low | low | low | low | high | low | low |
| ARIEL3 | low | low | low | low | low | low | low |
| OlympiAD | low | low | high | low | high | low | low |
| POLO | low | low | low | low | low | low | low |
| PAOLA-1 | low | low | low | unclear | low | low | low |
| PROfound | low | low | high | unclear | high | low | low |
